# Spherical Body Protein 2 truncated copy 11 as a specific *Babesia bovis* attenuation marker

**DOI:** 10.1186/s13071-018-2782-z

**Published:** 2018-03-12

**Authors:** Gina M. Gallego-Lopez, Audrey O. T. Lau, Wendy C. Brown, Wendell C. Johnson, Massaro W. Ueti, Carlos E. Suarez

**Affiliations:** 1 Department of Veterinary Microbiology and Pathology, College of Veterinary Medicine, Washington State University, Pullman, WA 99164-7040 USA; 2 The National Institute of Allergy and Infectious Diseases, the National Institutes of Health, Rockville, MD 20852 USA; 3 Animal Disease Research Unit, Agricultural Research Service, USDA, Pullman, WA 99164-6630 USA

**Keywords:** *Babesia bovis*, Spherical body protein, Transcription, PEXEL, Protein expression, Attenuation marker

## Abstract

**Background:**

Bovine babesiosis caused by *Babesia bovis* is a tick-borne hemoparasitic disease of global impact, and improved control is needed. In *B. bovis*, spherical body protein 2 (SBP*-2*) truncated copies 7, 9 and 11 (*sbp2t7*, *sbp2t9* and *sbp2t11*) gene transcripts were recently reported to be significantly upregulated in two geographically distinct attenuated *B. bovis* strains. In the present work, additional virulent and attenuated *B. bovis* strain pairs were compared in order to corroborate this finding.

**Results:**

Sequences of the *sbp2t7*, *sbp2t9* and *sbp2t11* genes were not fully conserved among geographically distinct *B. bovis* strains, and varied between 70.6–93.3% sequence identity in all three genes. Comparisons among transcript levels of the three *sbp2t* genes of distinct virulent-attenuated *B. bovis* strain pairs confirmed that upregulation of the *sbp2t11* gene was exclusively associated with an attenuated phenotype in the studied strain pairs. This rejects *sbp2t7* and *sbp2t9* as reliable attenuation markers. In addition, SBP2t11 protein was found to be significantly overexpressed in Texas attenuated *B. bovis* in comparison to the Texas virulent strain. Finally, *sbp2t11* was differentially expressed in blood stages of the parasite but undetectable in Texas strain kinetes.

**Conclusions:**

*Sbp2t11* is a strong candidate as a reliable attenuation marker for *B. bovis*, based on its consistent pattern of upregulation in four distinct attenuated strains when compared to their virulent parental strains. *Sbp2t11* may only have functional roles associated with erythrocyte infection. Identification of attenuation markers will lead to future research focused on the production of novel and safer subunit and genetically defined vaccines against *B. bovis*.

## Background

Bovine babesiosis, caused by *B. bovis*, is a tick-borne hemoparasitic disease that affects more than 500 million cattle annually worldwide [1]. Currently, the only effective strategy to alleviate economic losses and acute clinical pathologies is the use of live, attenuated vaccines. These live vaccines are produced from virulent parasites that undergo a series of quick passages in splenectomized calves [2]. However, such live vaccines have inherent risks and application issues, including reversion to virulence, contamination with other blood-borne pathogens, and the requirement of cold chain storage during transportation, as well as a high cost of production [3]. To improve upon this preventive care, a subunit vaccine is a preferred alternative, but none is currently available [4]. Crucial elements necessary to facilitate the development of subunit vaccines include a better understanding of the attenuation mechanisms or virulence determinants, and the identification of factors in the vaccine strains that contribute to protective immunity. Until a subunit vaccine can be developed, a stable live vaccine that cannot revert to virulence is important. Thus, if the factors involved in attenuation are identified, then a stable, live vaccine with no virulence reversion capability is possible.

At present, little is known about the identity of *Babesia* virulence factors. Attempts to decipher these factors resulted in recent reports that illustrate the alterations towards a reduced genome diversity in the attenuated strains obtained after repeated calf passages [5] as well as strain and phenotype-specific gene regulatory differences between virulent and derived attenuated strains [6]. Specifically, members of a *Babesia*-specific gene family, spherical body proteins (SBPs), were shown to be transcriptionally upregulated exclusively in the attenuated derivatives. Why these transcripts were upregulated in attenuated *B. bovis* remains unknown, but it is reasonable to hypothesize they may directly or indirectly be involved in the attenuation of virulence.

SBPs are localized within spherical body organelles in *B. bovis*, which are homologous to the dense granules found in other apicomplexans [7]. There are four spherical body proteins, SBP1-4, expressed in *B. bovis* erythrocyte stages. All contain signal peptides at the amino (N') terminus and are expected to be secreted proteins**.** The function of these SBPs is currently unknown; however, as all SBPs are released after merozoite invasion, they may contribute to growth, development, and/or survival of the intraerythrocytic parasites [7–12]. In addition, SBPs are hypothesized to participate in altered membrane permeability of infected erythrocytes, development of membrane protrusions, and infected-erythrocyte attachment to host endothelial cells at membrane protrusion sites [7]. SBP4 has been characterized as a specific marker for ELISA-based detection of different *B. bovis* strains [9, 12].

SBP2 is a protein conserved between different geographically isolates [8] and, unlike the other SBPs, consists of a 13-member *sbp2* gene family. In contrast to the larger 225 kDa SBP2 protein, the rest of the family members are truncated copies between 25–48 kDa. In truncated copies, a high sequence identity compared to SBP2 resides at the N’ terminus while the carboxyl terminus (C') identity varies.

The full-length *sbp2* (BBOV_II000740) and truncated copy 12 (*sbp2t12*, BBOV_II000680) genes are localized on chromosome 2, while the remaining eleven truncated copies are on chromosome 3. The truncated genes on chromosome 3 occur in three clusters of two, four, and five genes. Transcripts of all spherical body protein 2 truncated copies (*sbp2t*) were detected in blood stages [6]. The *sbp2t11* gene is expressed in *B. bovis* blood stages as a 30 kDa protein, which results in a 17 kDa product upon proteolytic cleavage mediated by a PEXEL-like motif (PLM) [13]. SBP2t11 is localized in a vesicle, likely the spherical body, and probably secreted to the infected intra-erythrocyte milieu [13].

Interestingly, transcripts of the spherical body protein 2 truncated copies (*sbp2t*) 7, 9 and 11 (BBOV_III006460, BBOV_III006500 and BBOV_III006540, respectively) were found to be upregulated by as much as five-fold in attenuated strains as compared to the virulent parental isolates [6]. In the current study, we extended the analysis of transcript levels of these three *sbp2t* genes using additional geographically distant strains to test the hypothesis that *sbp2* truncated gene members 7, 9 and 11 are attenuation markers for *B. bovis*.

## Methods

### In vitro propagation of *B. bovis* culture

*Babesia bovis* strains were maintained as in vitro blood culture in micro-aerophilus stationary-phase culture at 10% hematocrit using bovine erythrocytes as previously described [14]. These strains are geographically divergent virulent (vir) parental field isolates and their respective attenuated (att) derivatives were previously characterized [5]. They include Texas (Tx) and two Australian strains (T and D). Both Australian strains were provided by Dr Peter Rolls (Tick Fever Research Centre, Queensland, Australia).

### Genomic and complementary DNA synthesis

An in vitro *B. bovis* culture with approximately 25% parasitized erythrocytes (PPE) was used for the isolation of genomic DNA (gDNA) using Puregene Blood Core Kit C (Qiagen, MD, USA). Briefly, cells were centrifuged at 1048× *g* for 10 min at 4 °C and washed with cold phosphate buffered saline (PBS). The pellet was frozen at -80 °C and stored overnight to lyse erythrocytes and release merozoites. To extract gDNA, the frozen cell pellet was thawed on ice, washed in 10 ml of ice-cold PBS, centrifuged at 1509× *g* for 20 min at 4 °C and suspended in 500 μl of Puregene Cell Lysis Buffer (Qiagen). Two microliters of RNaseA solution was added and incubated at 37 °C for 20 min followed by the addition of 25 μl of 20 mg/ml proteinase K and 1 μl of 20 mg/ml glycogen. The mixture was incubated overnight at 56 °C. Two hundred microliters of protein precipitation solution (Qiagen) was added and centrifuged at 8900× *g* for 5 min. Five hundred microliters of 100% isopropanol at room temperature (RT) was added to the supernatant and mixed by inverting, followed by incubation at RT for 10 min and subsequent centrifugation at 17,530× *g* for another 10 min at 4 °C. The pellet was washed in 70% ethanol, allowed to air dry and suspended in 25 μl of DNA hydration buffer (Qiagen).

For the generation of cDNA, in vitro *B. bovis* cultures with 25% PPE were used for Trizol RNA isolation (Ambion, MA, USA). *Babesia bovis* cultures were centrifuged and the pellets suspended in 3 ml of Trizol. Total RNA was isolated as indicated by the manufacturer (J.T. Baker, PA, USA). Total RNA was suspended in 25 μl of diethylpyrocarbonate-treated water. Total RNA was treated with Turbo DNA free-DNase (Ambion). Reverse transcriptase-polymerase chain reaction (RT-PCR) was carried out for cDNA synthesis using random hexamers in the RT reaction (Retroscript, Ambion). cDNA from Tx_vir_ *B. bovis* kinetes was prepared as previously published [15].

### Analysis of transcriptional profile *sbp2t11* in kinete and blood stages

cDNA from Tx_vir_ *B. bovis* kinete and blood stage parasites was used to amplify a fragment of *sbp2t11* and the control gene ubiquitin by PCR. Sets of primers were designed to amplify a specific 155 bp fragment of *sbp2t11* and a 393 bp fragment from the ubiquitin gene BBOV_IV003190 (see Additional file 1: Table S1) at 1 cycle at 95 °C for 10 min; 35 cycles of 95°C for 10 s, 50 or 55 °C for 30 s, 72 °C for 30 s and finally 1 cycle at 72 °C for 5 min as reported previously [15] . Amplicons were cloned and sequenced as previously published [15].

### Identification of *sbp2t7*, *9* and *11* in Australian T and D strains

Degenerate primers were designed using MacVector v.11.1, NCBI Blast and known *sbp*2 truncated copy 7, 9 and 11 gene sequences from the Tx strains, to amplify and clone the three orthologous genes from the Australia T and D strain pairs (see Additional file 1: Table S1). Amplifications were carried out using the following PCR conditions: 1 cycle at 95 °C for 180 s; 39 cycles of 95 °C for 30 s, 54 °C for 30 s, 72 °C for 120 s and finally 1 cycle at 72 °C for 300 s (Sigma-Aldrich RedTaq reaction mix, MO, USA). The amplicons were gel purified, cloned using pCR®4-TOPO® vector system (Invitrogen, MA, USA) and sequenced (Qiagen Miniprep and Eurofins SimpleSeq DNA sequencing kit, Thermo Fisher Scientific, NV, USA). Subsequent strain-specific primers were designed to amplify the full-length of *sbp2t7*, *sbp2t9* and *sbp2t11* from cDNA of the Australian *B. bovis* strains. Validation of correct amplification was conducted by cloning and sequencing. Full-length of *sbp2t7*, *sbp2t9* and *sbp2t11* from the cDNA of Australian *B. bovis* strain pairs (T and D) were reported under the GenBank accession numbers MG430176, MG430177 and MG430178, respectively.

### Quantitative polymerase chain reaction (QPCR)

QPCR of *sbp*2*t7*, *sbp*2*t9* and *sbp*2*t11* was carried out as previously described [6]. Briefly, sybr green supermix (Biorad, CA, USA), specific primers and 70 ng cDNA was used for qPCR at 1 cycle at 95 °C for 10 min; 39 cycles of 95 °C for 10 s, 57.5 °C for 30 s, 72 °C for 30 s and finally 1 cycle at 72 °C for 5 min and 55 °C for 1 min. Expected amplicons were between 155 and 200 bp. Three replicates per strain were used. To normalize the reactions, amplification of BBOV_II004820 which encodes for a single copy topoisomerase II gene was also set up using the same template source. The transcript level of topoisomerase II remains the same in the virulent and attenuated *B. bovis* strain pairs [6]. Final data are represented as cycle threshold ratio (CT ratio) rather than the actual CT values. Additional file 1: Table S2 shows all qPCR primer sequences.

### Quantitation of SBP2t11 protein expression by densitometry and ELISA

Three biological replicates of Tx_vir_ and Tx_att_ *B. bovis* protein lysates were prepared as reported previously [13] and used in three independent western blot analyses [13]. Band intensities were analyzed using the Alphaimager system and software (Cell Biosciences, CA, USA). The 17 kDa band intensity was normalized to the merozoite surface antigen 1 (MSA-1) protein expression using MSA-1-specific monoclonal antibody (mAb) BABB35 [16]. Tx_vir_ and Tx_att_ *B. bovis* protein lysates with equal percent parasitized erythrocytes (PPE) were used in an indirect ELISA. Flat bottomed plates (Immulon-thermo, MA, USA) were coated overnight with 0.02 μg/μl of protein lysates in coating buffer (75 mM sodium carbonate and 16 mM sodium bicarbonate, pH 9.4). Plates were blocked for 1 h with 1× PBS, 0.05% Tween 20 and 5% bovine serum albumin (BSA). Fifty microliters of anti-SBP2t11 affinity purified antibody [13] or anti-MSA-1 (BABB35) at a dilution of 1:50 was incubated in the plates for 1 h. Washing was performed in an automated washing machine with 1× PBS, 0.05% Tween 20. The secondary antibodies used were goat anti-rabbit or goat anti-mouse IgG conjugated to horseradish peroxidase HRP (KPL 474-1516) at a dilution of 1:500 and plates were incubated for 1 h and washed. To develop the enzymatic reaction, 100 μl of 3,3',5,5'-tetramethylbenzidine (KPL 5300-01) was added to each well and the reaction terminated by adding 100 μl/well of stop solution (KPL 50-85-05). Results were read at 450 nm. Results are presented as the expression level of SBP2t11: MSA-1.

### Statistical analysis

Expression values represented as cycle thresholds were normalized to the housekeeping gene, DNA topoisomerase II (putative). The final data were plotted as CT ratio where a lower ratio value corresponds to higher expression. Upregulation of *sbp2t* genes was statistically significant if *P* < 0.01 using a one-way ANOVA with Bonferroni *post-hoc* analysis (GraphPad Prism v.6.0a). The expression levels of SBP2t11:MSA-1 represent the mean of three independent samples. Standard errors of the mean (SEM) are indicated. Statistically significant differences were analyzed at *P* < 0.05 using a Student’s t-test (GraphPad Prism v.6.0a).

## Results and discussion

### The *sbp2t11* gene is consistently upregulated among distinct attenuated *B. bovis* strains

We used two additional distinct *B. bovis* virulent and attenuated strain pairs to confirm the previously observed differential expression of the *sbp2t7*, *sbp2t9* and *sbp2t11* genes in attenuated strains [6]. These strain pairs originated in Australia where T_vir_ and D_vir_ were virulent field isolates and T_att_ and D_att_ were their corresponding attenuated derivatives [17–19]. Attenuation of these two strains was achieved using a similar rapid in vivo passage method as previously described [5, 17]. These strain pairs were chosen for their geographically distant isolation as compared to those from North and South America.

Sequence comparisons revealed numerous size and sequence polymorphisms in *sbp2t7*, *sbp2t9* and *sbp2t11* genes (see Additional file 2: Figure S1, Additional file 3: Figure S2, Additional file 4: Figure S3) among Tx and both Australian strain pairs (T and D). Specifically, the *sbp2t7* gene in the Australian virulent and attenuated strains is 864 bp long (see Additional file 2: Figure S1) with 93.3 and 92.1% identity at the nucleotide and protein levels, respectively, as compared to the Tx strain (see Additional file 1: Table S3). The *sbp2t9* gene in both Australian virulent and attenuated strains is 798 bp long. This gene has 70.6 and 64.3% identity at the nucleotide and protein levels, respectively, to the Tx strain gene (see Additional file 3: Figure S2 and Additional file 1: Table S3).

The *Sbp2t11* gene in virulent and attenuated T and D strains is 837 bp long with 86.7 and 84.7% identity at the nucleotide and protein levels, respectively, to the Tx strain (see Additional file 4: Figure S3 and Additional file 1: Table S3). In addition to the protein sequence changes derived from insertions and deletions in the DNA sequences, non-synonymous single nucleotide polymorphism (SNP) mutations were present (see Additional file 1: Table S3).

**Fig. 1:**
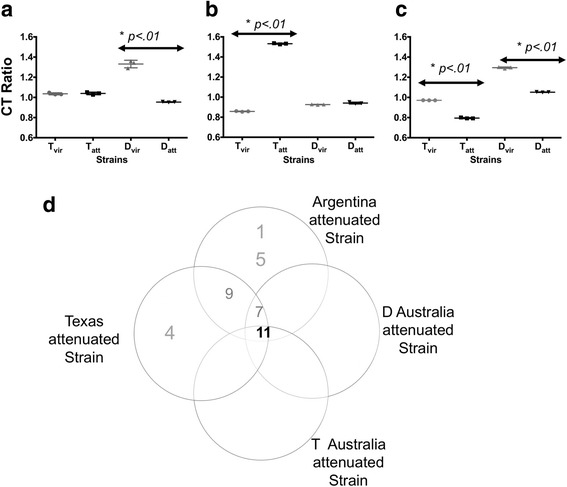
*Sbp2t11* is an attenuation marker upregulated in blood stages. Quantitative PCR to determine transcript levels of *sbp2t7*, *sbp2t9*, *sbp2t11* genes in T and D Australian strains. Transcript levels of (**a**) *sbp2t7*, (**b**) *sbp2t9* and (**c**) *sbp2t11* were measured and compared between virulent (grey) and attenuated (black) *B. bovis* Australian strains (T and D). Experiments were done in triplicates. * represents statistically significant difference. **d** Summary of upregulation of *sbp2t* transcripts in attenuated strains of *Babesia bovis*. Numbers indicate the corresponding *sbp2t* gene. 1, *sbp2t1*; 4, *sbp2t4*; 5, *sbp2t5*; 7, *sbp2t7*; 9, *sbp2t9*; 11, *sbp2t11*. *Abbreviations*: CT, cycle threshold; Vir, virulent; Att, attenuated

The transcriptional levels of *sbp2t7*, *sbp2t9* and *sbp2t11* from in vitro cultured blood stages parasites were compared among the virulent and derived attenuated strains using qPCR under the same conditions as reported previously [6]. The results indicated that *sbp2t7* was not differentially regulated between T_vir_ and T_att_ strains while it was significantly upregulated in D_att_ strain (Fig. 1a). *Sbp*2*t9* was significantly upregulated in T_vir_ strain but was not different in transcript levels in the D strain pairs (Fig. 1b). In contrast, transcript levels of *sbp2t11* were significantly upregulated in both Australian T_att_ and D_att_ as compared to the parental strains (Fig. 1c). The *sbp2t11* transcription analysis corroborates previously published data using Argentina and Texas *B. bovis* strains [6] and further confirms that upregulation of *sbp2t11* is a shared transcript signature among attenuated strains (Fig. 1d). Thus, the additional comparative expression analysis of new *B. bovis* strain pairs performed in this study allowed rejecting *sbp2t7* and *sbp2t9* as attenuation markers for this parasite. The *sbp2t11* gene remains as the only attenuation marker so far known for *B. bovis*. Figure 1d summarizes the transcriptional analysis results found in Texas, Argentina [6] and the Australian strains reported in this study.

### Expression of the SBP2t11 protein

**Fig. 2:**
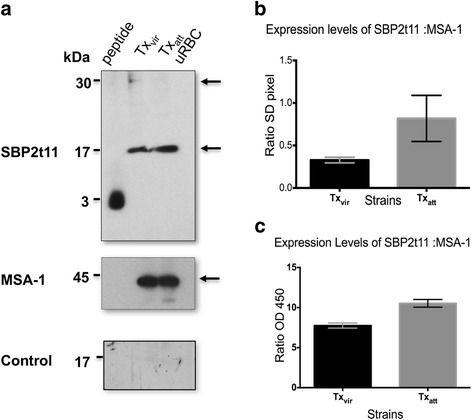
Protein expression quantitation of SBP2t11 by densitometry and ELISA. **a** SBP2t11 expression in Tx_vir_ and Tx_att_ strains. Controls include the peptide used to generate antibody, uninfected red blood cells (uRBC) and as loading control MSA-1 protein expression. Pre-immune rabbit sera (1:1000) were used as a control in Panel (**a**). **b** Expression levels of SBP2t11:MSA-1. The ratios were calculated by densitometry analysis performed on the 17 kDa-SBP2t11 and the 42 kDa MSA-1 bands. Three biological replicates of protein lysates of Tx_vir_ and Tx_att_ *B. bovis* strains were used to run three independent western blots. Band intensity was measured and analyzed using Alphaimager system and software (Cell Biosciences). The intensity of the 17 kDa bands was normalized to the MSA-1 protein expression. **c** Expression levels of SBP2t11: MSA-1 by indirect ELISA. The levels of expression of SBP2t11 and MSA-1 in protein lysates from in vitro cultures were tested using an anti-SBP2t11 and anti-MSA-1 antibodies in Tx_vir_ and Tx_att_ by ELISA. Expression of SBP2t11 was normalized to the MSA-1 protein expression. The graphs represent the mean of three independent samples and the SEM. * represents statistically significant difference at *P* < 0.05 using the Student’s t-test by GraphPad Prism v.6

We previously reported protein expression of SBP2t11 in the Tx *B. bovis* strain by conducting western blot analysis using an affinity-purified polyclonal anti-SBP2t11 antibody where we demonstrated both the expression of SBP2t11 and its cleavage from the 30 kDa full-length protein to a predominant C-terminal 17 kDa protein, determined by the recognition of a PEXEL-like motif (PLM) [13]. The anti-SBP2t11 antibody was produced against a specific small synthetic peptide at the carboxyl terminus designed because of the high sequence identity shared between SBP2 truncated protein family members at the amino terminus (see Additional file 5: Figure S4 and Additional file 1: Table S4). In this study, we performed quantitative protein analysis to investigate if differences in *sbp2t11* transcription levels among various attenuated and virulent strains are extended at the protein level using western blot and ELISA-based analyses. The western blot analysis data first confirmed that SBP2t11 was detectable in attenuated and virulent parasites (Fig. 2a). The ratios among SBP2t11:MSA-1 expression levels were estimated by densitometry analysis performed on the 17 kDa bands on the membranes. We were unable to repeat protein quantitation using the 30 kDa full-size SBP2t11 because signals were too weak (Fig. 2b). Based on densitometry values obtained for the 17 kDa bands, there was no statistical difference in the protein level between attenuated and virulent strain pair (Fig. 2b). In contrast, SBP2t11:MSA-1 expression levels among Tx_att_ and Tx_vir_ strains, obtained upon analysis using quantitative ELISA, using the same antibodies, demonstrated that the attenuated strain expresses a significantly higher amount of SBP2t11 protein than the virulent strain (Fig. 2c). Importantly, the ELISA allows the measuring of anti-SBP2t11 binding to the cleaved and full-length versions of SBP2t11, rendering the comparison using the ELISA method a more inclusive approach to detect SBP2t11. Collectively, the data show that the overexpression of the *sbp2t11* gene is manifested at both the transcriptional and translational levels in Tx_att_ strain, supporting the notion that *sbp2t11* is an attenuation marker for *Babesia bovis*. The biological significance of this observation goes beyond the scope of this study and will be further addressed in ongoing experiments. The correlation between upregulation and overexpression of *sbp2t11* in attenuated strains could be present in different geographically attenuated strains but specific antibodies are needed to confirm this phenomenon.

The antibody raised against the SBP2t11 protein of the Tx strain does not react in immunoblots with any antigen derived from the Australian strains, likely due to sequence differences in the regions used to generate the antibody among these distinct strains (Additional file 1: Table S4). This outcome prevented assessing differences in the level of protein expression of the attenuated and virulent Australian strains in this study using immunoblots.

### Transcriptional profile of *sbp2t11* in distinct *B. bovis* life stages

**Fig. 3:**
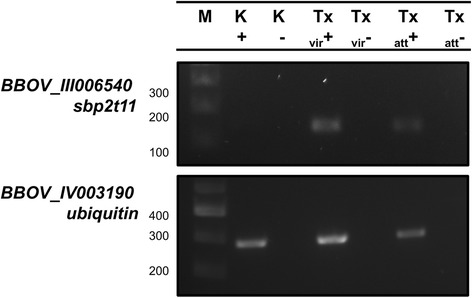
Expression of *sbp2t11* gene in kinete and blood stages of *B. bovis*. *sbp2t11* was amplified from cDNA of Tx_vir_ kinetes and Tx_vir_/Tx_att_ blood stages of *B. bovis*. BBOV_IV003190, a ubiquitin gene was used a control. tRNA not reverse transcribed was also used as control. (+) cDNA; (-) RNA represents not reverse-transcribed total RNA. *Abbreviations*: K, kinetes; Tx_vir_, virulent Texas strain; Tx_att_, attenuated Texas strain; M, marker 1 kb molecular weight ladder

The transcriptional profile of *sbp2t11* in *B. bovis* was analyzed in Tx strain kinete and blood stage parasites using RT-PCR on total RNA extracted from *B. bovis* kinetes present in the hemolymph of ticks infected with *B. bovis* and in vitro cultured blood stage parasites. Whereas the control ubiquitin transcript was detectable in all samples at relatively similar intensities, *sbp2t11* was only detected in blood stages of both Tx_vir_ and Tx_att_ (Fig. 3). Importantly, the RT-PCR products were sequenced to confirm their specificity. The data showed that *sbp2t11* is differentially expressed exclusively in Tx blood stages which suggests that its function is mainly associated with the intraerythrocytic life-cycle of the parasite.

## Conclusions

In this study, we hypothesized that *sbp2* truncated gene members 7, 9 and 11 were attenuation markers for *B. bovis*. Comparative *in silico* analysis showed that there are significant size variations and SNPs present in the three *sbp*2 truncated genes among different geographically divergent *B. bovis* strains. Also, we found strain-specific regulatory transcript patterns in *sbp2t7* and *sbp2t9* (Fig. 1a and b) which rejects *sbp2t7* and *sbp2t9* as reliable attenuation markers. The observation that only *sbp2t11* was consistently upregulated at the transcriptional level in blood stages of all evaluated attenuated strains places *sbp2t11* as a strong candidate for a reliable/consistent attenuation marker (Fig. 1d). In addition, overexpression of SBP2t11 in a Tx_att_ strain as compared to Tx_vir_ strain was confirmed at the protein level. The transcriptional profile of *sbp2t11* demonstrated that expression of this gene is downregulated in Tx strain kinetes, suggesting a functional role of *sbp2t11* exclusively in *B. bovis* blood stages. Further studies including the phenotypical characterization of a *B. bovis sbp2t11* knockout model system and mutants overexpressing SBP2t11 combined with in vivo experiments will help us to elucidate the functional significance of SBP2t11 in attenuation.

## Additional files

Additional file 1: **Table S1.** Primers used to amplify *sbp2t7*, *sbp2t9* and *sbp2t11* in Australian T and D strains. Sequence of primers designed and used to amplify target genes from cDNA as indicated in methods. Tm and amplicon size are indicated. **Table S2.** Primers used for quantitative PCR of *sbp2t7*, *sbp2t9* and *sbp2t11* in Australian T and D strains. The sequence of primers designed and used for qPCR of each gene analyzed as indicated in methods. Amplicons were validated by cloning and sequencing. Tm and amplicon size are indicated. **Table S3.** Nucleotide comparison of *sbp2t7*, *sbp2t9* and *sbp2t11* between Texas and Australian strains. *Sbp2t7*, *sbp2t9* and *sbp2t11* in T and Dixie Australian *B. bovis* strains were reported in GenBank with accession numbers MG430176, MG430177 and MG430178, respectively. These sequences were compared with the reference Texas strain of *B. bovis* by MacVector 12.0.5. Base pair (bp), nucleotide percent identity and amino acid percent identity are indicated. DOC. **Table S4.** Percentage identity between SBP2 and its 12 truncated proteins. Amino acid sequence alignment of SBP2 protein and its twelve truncated proteins (SBP2t) in Texas *Babesia bovis* strain were performed by MacVector 12.0.5. Identity scores are indicated in percentages. (DOCX 32 kb)

Additional file 2: **Figure S1.** Nucleotide alignment of *sbp2t7* gene in Australian and Texas *B. bovis*. (PDF 198 kb)

Additional file 3: **Figure S2.** Nucleotide alignment of *sbp2t9* gene in Australian and Texas *B. bovis*. (PDF 203 kb)

Additional file 4: **Figure S3.** Nucleotide alignment of *sbp2t11* gene in Australian and Texas *B. bovis*. (PDF 193 kb)

Additional file 5: **Figure S4.** Comprehensive amino acid sequence alignment of the SBP2 family members (*n* = 13). Members of the SBP2 proteins are highly conserved at the N’ terminus and more variable at the C’ terminus. Exact identity values are shown in Additional file 1: Table S4. Red boxes indicate the PEXEL-like motif (PLM) and number of amino acids of each protein. *Abbreviations*: T, truncated; SBP, spherical body protein. (PDF 682 kb)

## Abbreviations

Att: attenuated
PLM: PEXEL-like motif
PPE: percent parasitized erythrocytes
qPCR: quantitative polymerase chain reaction
RBC: red blood cells
*sbp2t*: spherical body protein 2 truncated copies
SNP: single nucleotide polymorphism
T/D: T and Dixie Australian *B. bovis* strains
Tx: Texas *B. bovis* strain
Vir: virulent
