# Supplementary material for: Spherical Body Protein 2 truncated copy 11 as a specific Babesia bovis attenuation marker
Source: Parasit Vectors. 2018 Mar 12;11:169. doi: 10.1186/s13071-018-2782-z (PMC5848574; doi:10.1186/s13071-018-2782-z)
Supplement: Supplementary file 1 — Table S1. Primers used to amplify sbp2t7, sbp2t9 and sbp2t11 in Australian T and D strains. Sequence of primers designed and used to amplify target genes from cDNA as indicated in methods. Tm and amplicon size are indicated. Table S2. Primers used for quantitative PCR of sbp2t7, sbp2t9 and sbp2t11 in Australian T and D strains. The sequence of primers designed and used for qPCR of each gene analyzed as indicated in methods. Amplicons were validated by cloning and sequencing. Tm and amplicon size are indicated. Table S3. Nucleotide comparison of sbp2t7, sbp2t9 and sbp2t11 between Texas and Australian strains. Sbp2t7, sbp2t9 and sbp2t11 in T and Dixie Australian B. bovis strains were reported in GenBank with accession numbers MG430176, MG430177 and MG430178, respectively. These sequences were compared with the reference Texas strain of B. bovis by MacVector 12.0.5. Base pair (bp), nucleotide percent identity and amino acid percent identity are indicated. DOC. Table S4. Percentage identity between SBP2 and its 12 truncated proteins. Amino acid sequence alignment of SBP2 protein and its twelve truncated proteins (SBP2t) in Texas Babesia bovis strain were performed by MacVector 12.0.5. Identity scores are indicated in percentages. (DOCX 32 kb) [file 13071_2018_2782_MOESM1_ESM.docx]

**Table S1. Primers used to amplify *sbp*2t7, 9 and 11 in Australian T and D strains and *sbp2t11* in Tx strain.**

| **Primer names** | **Sequences (5’-3’)** | **Tm (°C)** | **Amplicon sizes (bp)** |
| --- | --- | --- | --- |
| sbp2t7FAus | ATGGAACTTGCAAGGCGTAACAACG | 60.2 | 854 |
| sbp2t7RAus | CTTCTTTGGACTCCTTACCTTCATCTACAC | 58.2 |  |
| sbp2t9FAus | ATGGAAGTAGCAAGGCGTAACAAC | 57.5 | 966 |
| sbp2t9RAus | TTACCAAAATTTGTACCATGGCTTG | 54.5 |  |
| sbp2t11FAus | ATGGAACTAGCAAGGCGTAACAACG | 59.4 | 838 |
| sbp2t11RAus | TTACCAAAATTTGTACCATGGCTTG | 54.5 |  |
| sbp2t11FTx | ACCTAGCCAATGCTCTTGAGG | 55 | 155 |
| sbp2t11RTx | GTTGTTCCTGCTCCTCCCAT | 55 |  |
| ubiquitinF | ATGCAGATTTTCGTTAAAAC | 50 | 393 |
| ubiquitinR | GCTACCACCCTTAATCTTC | 50 |  |

Sequence of primers designed and used to amplify target genes from cDNA as indicated in methods. Tm and amplicon size are indicated *Sbp2t*, spherical body protein 2 truncated copies.

**Table S2. Primers used for quantitative PCR of *sbp*2t7, 9 and 11 in Australian T and D strains**

| **Primer names** | **Sequences (5’-3’)** | **Tm (°C)** | **Amplicon sizes (bp)** |
| --- | --- | --- | --- |
| qPCR-sbp2t7FAus | TGTAAAAGTTGCCCACGCC | 56.3 | 191 |
| qPCR-sbp2t7RAus | TACCTGTCAAACCAGTTCGG | 54.6 |  |
| qPCR-sbp2t9FAus | TGGAAGTAGCAAGGCGTAACA | 56.5 | 112 |
| qPCR-sbp2t9RAus | TTCGAAGGACGACCACCTTG | 55.1 |  |
| qPCR-sbp2t11FAus | AGTGGATTGTTGGTGCCGTG | 58.3 | 200 |
| qPCRsbp2t11RAus | TGCAGATGGGTCGTGTGG | 57.8 |  |

Sequence of primers designed and used for qPCR of each gene analyzed as indicated in methods. Amplicons were validated by cloning and sequencing. Tm and amplicon size are indicated. *Sbp*2t, spherical body protein 2 truncated copies

**Table S3.  Nucleotide comparison of *sbp*2t7, 9 and 11 between Texas and Australian strains.**

| **Genes** | **T_x_ (BP)** | **T/D (bp)** | **Nucleotides**  **Percent Identity** | **Protein Percent Identity** |
| --- | --- | --- | --- | --- |
| *sbp2t7* | 873 | 864 | 93.3 | 92.1 |
| *sbp2t9* | 876 | 798 | 70.6 | 64.3 |
| *sbp2t11* | 816 | 837 | 86.7 | 84.7 |

*Sbp2t7, sbp2t9* and *sbp2t11* in T and Dixie Australian *B. bovis* strains were reported in GenBank accession number MG430176, MG430177 and MG430178, respectively. These sequences were compared with the reference Texas strain of *B. bovis* by MacVector 12.0.5. Base pair (BP), nucleotide percent identity and amino acid percent identity are indicated. *Sbp2t*, spherical body protein 2 truncated copies; Tx, Texas; T/D, Australia T and D strains

**Table S4. Percentage identity between SBP2 and its 12 truncated proteins**

**.**

| **Identity Scores (%)** |
| --- |

| Protein | SBP2 | SBP2t1 | SBP2t2 | SBP2t3 | SBP2t4 | SBP2t5 | SBP2t6 | SBP2t7 | SBP2t8 | SBP2t9 | SBP2t10 | SBP2t11 | SBP2t12 |
| --- | --- | --- | --- | --- | --- | --- | --- | --- | --- | --- | --- | --- | --- |
| SBP2  BBOV_II000740 | 100 | 3.2 | 3 | 3.4 | 2.9 | 3.5 | 2.3 | 3.5 | 2.7 | 2.7 | 3.4 | 2.9 | 3.7 |
| SBP2t1  BBOV_III005600 | 4.6 | 100 | 22.7 | 23.2 | 52.7 | 17.1 | 18.6 | 23.1 | 51.7 | 50.7 | 19.5 | 54.2 | 16.1 |
| SBP2t2  BBOV_III005630 | 5.1 | 35.5 | 100 | 52.8 | 28.2 | 47.6 | 20.1 | 62.1 | 27.4 | 27.7 | 49.7 | 28.1 | 18 |
| SBP2t3  BBOV_III005790 | 5.4 | 36.5 | 59.9 | 100 | 23.5 | 33.6 | 31.9 | 56.7 | 24.8 | 24.8 | 68.2 | 24.9 | 20.8 |
| SBP2t4  BBOV_III005830 | 4.2 | 62.5 | 39.4 | 35.8 | 100 | 18.5 | 19.6 | 26.7 | 57.3 | 56.1 | 21.5 | 65.7 | 14.9 |
| SBP2t5  BBOV_III005840 | 6.3 | 26.9 | 54.8 | 39.4 | 27.1 | 100 | 15.8 | 42.7 | 21.9 | 21.9 | 34.3 | 20.3 | 14 |
| SBP2t6  BBOV_III005860 | 4.3 | 30.7 | 29.3 | 41.1 | 30.1 | 24.1 | 100 | 24.6 | 21.8 | 21.8 | 27.1 | 20.1 | 15 |
| SBP2t7  BBOV_III006460 | 5.2 | 37.7 | 70.8 | 64.3 | 39.4 | 49.4 | 35.3 | 100 | 26.2 | 26.2 | 71.4 | 26.7 | 18.3 |
| SBP2t8  BBOV_III006480 | 4.6 | 65.6 | 38.8 | 37.1 | 65.3 | 30.5 | 35.5 | 39.1 | 100 | 97.9 | 24.1 | 73.3 | 15.7 |
| SBP2t9  BBOV_III006500 | 4.6 | 64.3 | 39.1 | 37.1 | 63.9 | 30.8 | 35.5 | 39.1 | 97.9 | 100 | 24.1 | 71.8 | 15.7 |
| SBP2t10  BBOV_III006520 | 5.1 | 32.8 | 56 | 75.2 | 33.5 | 39.6 | 39 | 74.5 | 35.7 | 36.1 | 100 | 23.5 | 20.7 |
| SBP2t11  BBOV_III006540 | 4.4 | 64.8 | 40.3 | 37.7 | 73.4 | 29.6 | 32.1 | 40.3 | 80.7 | 79 | 36.3 | 100 | 15.5 |
| SBP2t12  BBOV_II000680 | 6.9 | 24.7 | 27.6 | 32.9 | 22.6 | 24.5 | 26.4 | 29 | 24.9 | 24.9 | 33.2 | 23.7 | 100 |

Amino acid sequence alignment of SBP2 protein and its twelve truncated proteins (SBP2t) in Texas *Babesia bovis* strain were performed by MacVector 12.0.5. Identity scores are indicated in percentages.
