# Supplementary figures and images for: Spherical Body Protein 2 truncated copy 11 as a specific Babesia bovis attenuation marker
Source: Parasit Vectors. 2018 Mar 12;11:169. doi: 10.1186/s13071-018-2782-z (PMC5848574; doi:10.1186/s13071-018-2782-z)

*sbp2t7* Nucleotide Alignment in Texas and Australian *Babesia bovis* strains

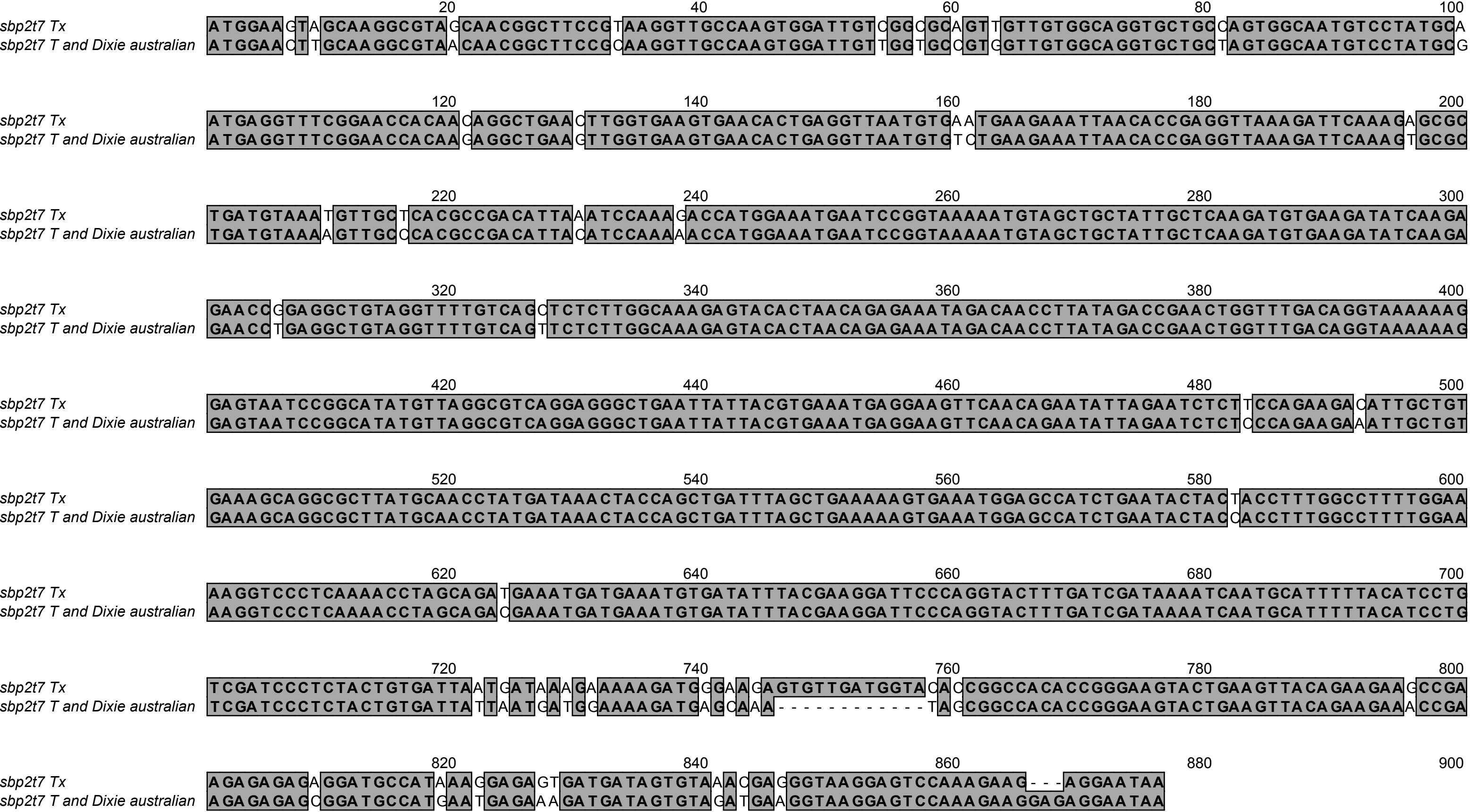

Supplement: Supplementary file 2 — Figure S1. Nucleotide alignment of sbp2t7 gene in Australian and Texas B. bovis. (PDF 198 kb) [file 13071_2018_2782_MOESM2_ESM.pdf]

sbp2t11 Nucleotide Alignment in Texas and Australian Babesia bovis strains

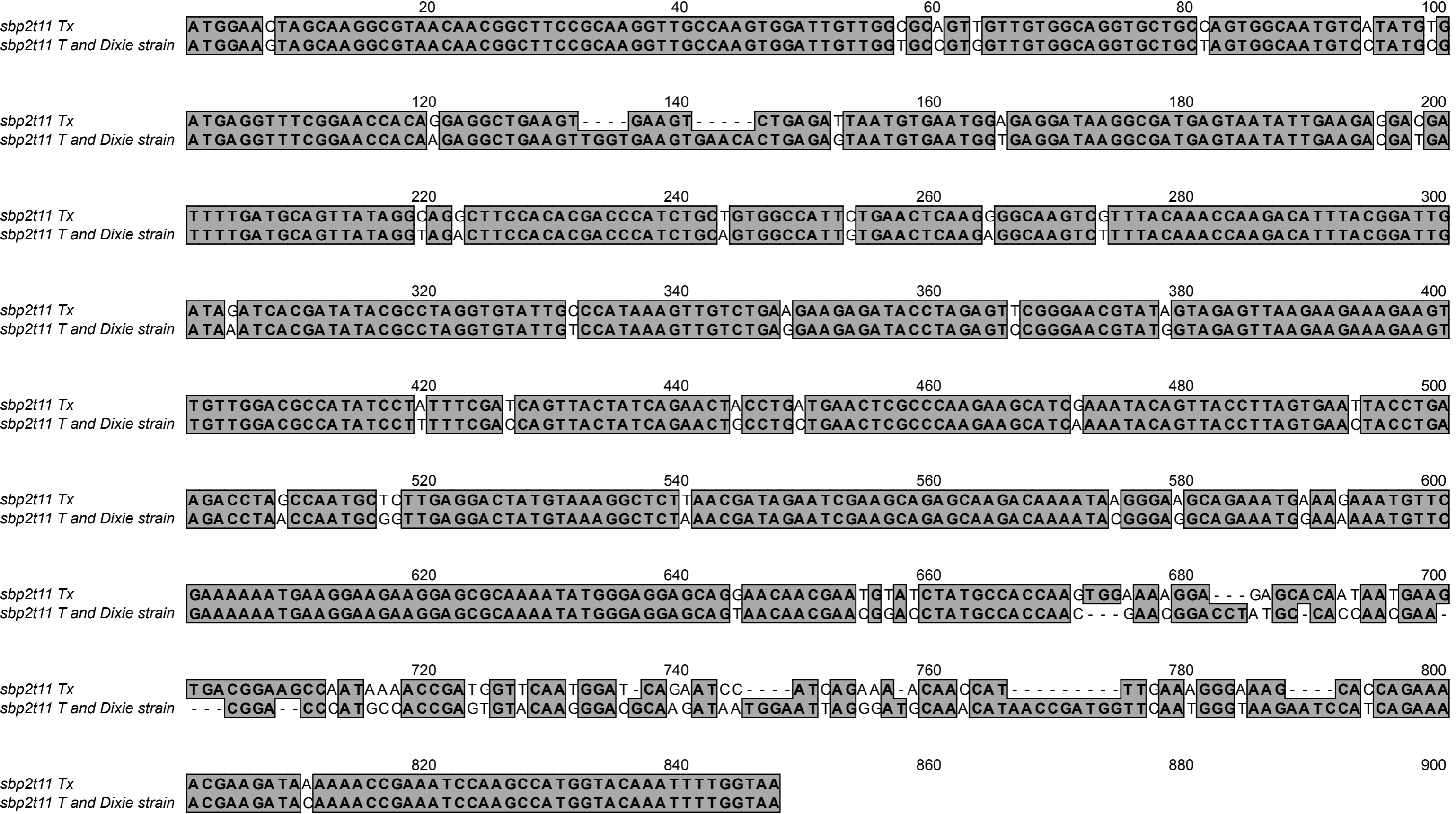

Supplement: Supplementary file 4 — Figure S3. Nucleotide alignment of sbp2t11 gene in Australian and Texas B. bovis. (PDF 193 kb) [file 13071_2018_2782_MOESM4_ESM.pdf]
