# Supplementary material for: Spherical Body Protein 2 truncated copy 11 as a specific Babesia bovis attenuation marker
Source: Parasit Vectors. 2018 Mar 12;11:169. doi: 10.1186/s13071-018-2782-z (PMC5848574; doi:10.1186/s13071-018-2782-z)
Supplement: Supplementary file 3 — Figure S2. Nucleotide alignment of sbp2t9 gene in Australian and Texas B. bovis. (PDF 203 kb) [file 13071_2018_2782_MOESM3_ESM.pdf]

sbp2t9 Nucleotide Alignment in Texas and Australian Babesia bovis strains

```
                             20        40        60        80       100
sbp2t9 Tx                    ATGGAACTAGCAAGGCGTAACAACGGCTTCCGCAAGGTTGCCAAGTGGATTGCTGGTGCCCTTGTTGTGGCAGGTGCTGCCAGCGGAAATGTCCTATGTG
sbp2t9 T and Dixie strain     ATGGAAGTAGCAAGGCGTAACAACGGCTTCCGCAAGGTTGCCAAGTGGATTGTTGGTGCCCTTGTTGTGGCGAGTGCTAGCAGTGGCATTGTCCAAGGTG
                             ATGGAASTAGCAAGGCGTAACAACGGCTTCCGCAAGGTTGCCAAGTGGATTGYTGGTGCCCTTGTTGTGGCRRGTGCTRSCAGYGGMAWTGTCCWAKGTG

                            120       140       160       180       200
sbp2t9 Tx                    ATGAGGTTTCGGAACCACAGGAGGCTGAAG---------TGAAGTCTGAGATTAATGTGAATGGAGAGGATAAGGCGATGAGTAATATTGAGAACGATGA
sbp2t9 T and Dixie strain     GTCGTCCTTCGAAACCAAAACAGGCCGAAGCTAATGAGGTTAAGTCTAAAATTAATGTGATTTGAAGAAAAAAAGGAGATGCCTAAAATTGGGGACGATGA
                             RTSRKSYTTCGRAACCAMARSAGGCYGAAGCTAATGAGGTKAAGTCTRARATTAATGTGAWTGRAGARRAWAAGGMGATGMSTAAWATTGRGRACGATGA

                            220       240       260       280       300
sbp2t9 Tx                    TTTTGAAGCAATTATAGGCAGACTTCCACACGACCCTGCTGCAGTGGCCATTGTGAACTCAAGGGGCAAGTCCTTTACAAACCAAGACATTTACGGATTG
sbp2t9 T and Dixie strain     GTTTCAAGCAGTTATAGGCAGACTTCCACATGACCCCGCTGCAGTGGCGGTTGTGAACTCAAGAGGCAAGTCTTTTACAAACCAAGACATTTACGGATTG
                             KTTTSAAGCARTTATAGGCAGACTTCCACAYGACCCYGCTGCAGTGGCSRTTGTGAACTCAAGRGGCAAGTCYTTTACAAACCAAGACATTTACGGATTG

                            320       340       360       380       400
sbp2t9 Tx                    ATCGATCACGATATACGCCTAGGTTTATTGTCCATAACGTTGACTGAAGAAGAGATACCAAGCGTCCGGGAAGGTATAGTAGAGTTAAGAAGACAGAAAT
sbp2t9 T and Dixie strain     ATAGATCATAATCTCTACGTTGGCTTTATGTGGAAAAAGATACCTGATGAAAATAAACCAGAAATCCGGGAACGTATAGTAGAGTTAAGAAGAAAGAATT
                             ATMGATCAYRATMTMYRCSTWGGYTTWWTGTSSAWAAMGWTRMCTGAWGAARAKAWACCARRMRTCCGGGAASGTATAGTAGAGTTAAGAAGAMAGAAWT

                            420       440       460       480       500
sbp2t9 Tx                    TATTGGACGCCATATCCTTTTTCGATCAGCTACTATCAGAACTACCTGCTGAACTCGCCCAAGAAGCATCAAAGTACAGCTACCTTAGTGAACTACCTGA
sbp2t9 T and Dixie strain     TGTTGGACGAGATATCCTATTTCGACCAGCTACTATCAGAACTACCTGCTGATCTCGCCCAAGAAGCATCAAAATACAGTTACCTTAGTGAACTACCTGA
                             TRTTGGACGMSATATCCTWTTTCGAYCAGCTACTATCAGAACTACCTGCTGAWCTCGCCCAAGAAGCATCAAARTACAGYTACCTTAGTGAACTACCTGA

                            520       540       560       580       600
sbp2t9 Tx                    AGACCTAGCTAGTGCTCTTGACAAGTATGTCAAGGCTCTAAACGATAGAATCGAAAAAGAGCAAGACAAAATACGGGAGGCAGAAATGAAAAAAATGGTA
sbp2t9 T and Dixie strain     AGACCTAGCCGATGCTCTTGAGGACTATGTAAAGGCTCTTAACGATAGAATCGAAGCAGAACAAGACAAAATAAGGGAGAAACAAATAAAGAATTGG--
                             AGACCTAGCYRRTGCTCTTGASRASTATGTMAAGGCTCTWAACGATAGAATCGAARMAGARCAAGACAAAATAMGGGAGRMARMAAWKAAARAAWTGGTA

                            620       640       660       680       700
sbp2t9 Tx                    GCTAAATGGCGAAAACAAAGAGGAAGAGTAGACGAAGATGATGAATACGACGAAGAAGATGAAGAGGAGGACGAAGATGATCAACCCATAAAGAGCAAGA
sbp2t9 T and Dixie strain     ATGAAATGA--GAAACAAAGACTTAAAG-----GAAGCT--CCAA-AGGAACAAGGAGATCAAAAGGAACAAGTAGAT-------CCA-AAGGAACAAGG
                             RYKAAATGRCGRAAACAAAGASKWARAGTAGACGAAGMTGAYSAATASGAMSAAGRAGATSAARAGGARSAMGWAGATGATCAACCCATAARGARCAAGR

                            720       740       760       780       800
sbp2t9 Tx                    TCAGTGCAAAGAAAACAAGAAGATCACAGCAAGAGGAGGAAGAAGAGGACGAAGATGATCAACCCATAAAGAGCAAGATCAGTGCAAAGGACCAGAATCC
sbp2t9 T and Dixie strain     --AGATCAAAAGGAACAAGTAGATC--------CGAAGGAACAAGTAGA---------TCAA-----AAGGAACAAG-TAGATCCAAAGGAACAAG----
                             TCAGWKCAAARRAACAAGWAGATCACAGCAAGMGRAGGAASAAGWRGACGAAGATGATCAACCCATAARGARCAAGATMRRTSCAAAGGAMCARRATCC

                            820       840       860       880       900
sbp2t9 Tx                    ATCAGAAAACAACCATTTGAAAGGAAAAGCGCCAGAAAACGAAGATAAAAAGCCAAAATCCAAGCCATGGTACAAATTTTGGTAA
sbp2t9 T and Dixie strain     ----GAGAGCA-------GAACAAAAAA---------AGCAAGTCTAAAAA---AGGATTAAGGAAATGA---------------
                             ATCAGARARCAACCATTTGAAMRRAAAAGCGCCAGAAARCRARKMTAAAAAGCCARRATYMARGMMATGRTACAAATTTTGGTAA
```
